# Generalist care managers for the treatment of depressed medicaid patients in North Carolina: A pilot study

**DOI:** 10.1186/1471-2296-8-7

**Published:** 2007-03-05

**Authors:** Suzanne E Landis, Bradley N Gaynes, Joseph P Morrissey, Nina Vinson, Alan R Ellis, Marisa E Domino

**Affiliations:** 1 Department of Family Medicine, Mountain Area Health Education Center and University of North Carolina, Chapel Hill, NC, USA; 2 Department of Psychiatry, University of North Carolina, Chapel Hill, NC, USA; 3 Cecil G. Sheps Center for Health Services Research, University of North Carolina, Chapel Hill, NC, USA; 4 SAGE Partners, Inc. Asheville, NC, USA; 5 Department of Health Policy and Administration, School of Public Health, University of North Carolina, Chapel Hill, NC, USA

## Abstract

**Background:**

In most states, mental illness costs are an increasing share of Medicaid expenditures. Specialized depression care managers (CM) have consistently demonstrated improvements in patient outcomes relative to usual primary care (UC), but are costly and may not be fully utilized in smaller practices. A generalist care manager (GCM) could manage multiple chronic conditions and be more accepted and cost-effective than the specialist depression CM. We designed a pilot program to demonstrate the feasibility of training/deploying GCMs into primary care settings.

**Methods:**

We randomized depressed adult Medicaid patients in 2 primary care practices in Western North Carolina to a GCM intervention or to UC. GCMs, already providing services in diabetes and asthma in both study arms, were further trained to provide depression services including self-management, decision support, use of information systems, and care management. The following data were analyzed: baseline, 3- and 6-month Patient Health Questionnaire (PHQ9) scores; baseline and 6-month Short Form (SF) 12 scores; Medicaid claims data; questionnaire on patients' perceptions of treatment; GCM case notes; physician and office staff time study; and physician and office staff focus group discussions.

**Results:**

Forty-five patients were enrolled, the majority with preexisting depression. Both groups improved; the GCM group did not demonstrate better clinical and functional outcomes than the UC group. Patients in the GCM group were more likely to have prescriptions of correct dosing by chart data. GCMs most often addressed comorbid conditions (36%), then social issues (27%) and appointment reminders (14%). GCMs recorded an average of 46 interactions per patient in the GCM arm. Focus group data demonstrated that physicians valued using GCMs. A time study documented that staff required no more time interacting with GCMs, whereas physicians spent an average of 4 minutes more per week.

**Conclusion:**

GCMs can be trained in care of depression and other chronic illnesses, are acceptable to practices and patients, and result in physicians prescribing guideline concordant care. GCMs appear to be a feasible intervention for community medical practices and to warrant a larger scale trial to test their appropriateness for Medicaid programs nationally.

## Background

Given the substantial concern about the broad under-diagnosis and under-management of depression in primary care [1], most primary care treatment intervention strategies have aimed to improve outcomes through better detection and by increasing the likelihood of guideline concordant treatment in the acute phase [2]. These strategies have focused interventions at the system level, in studies such as the Patient Outcomes Research Team (PORT) for depression [3], the QuEST project [4], and the Depression Management Program for high utilizers [5], and also at the patient level [6-8].

Successful implementation of these strategies leads to improved outcomes of depressed primary care patients. A meta-analysis of randomized clinical trials of depression screening [9] demonstrated that overall, screening and physician feedback reduced the risk for persistent depression (summary relative risk, 0.87 [95% CI, 0.79 to 0.95]). Feedback alone is of questionable benefit; improvement in outcome is strongest in clinic systems in which screening and feedback are combined with system changes that help ensure adequate treatment and follow-up. These results led the U.S. Preventive Services Task Force to recommend screening adults for depression only in clinical practices that have systems in place to ensure accurate diagnosis, effective treatment, and coordinated follow-up [9].

These three elements – accurate diagnosis, effective treatment, and coordinated follow-up – are the core features of depression care management, a strategy in which a trained individual enhances the delivery of clinical services by assisting the physician with the identification, treatment, and closely monitored follow-up of primary care patients with depression. Such enhancement can help the primary care physician better address the competing clinical demands inherent in routine practice (e.g., acute vs. chronic illness care) [10]. Care managers who specialize only in depression have consistently shown improvements in patient outcomes relative to usual primary care in randomized controlled trials. Hunkeler reported a benefit of 57% response vs. 38% response at 6 months (p = 0.003)[11]. Use of such specialist care managers in collaborative care studies has resulted in improvements in remission at 6 months (44% vs. 31%, p = 0.05)[12] and treatment response at 4 months (70.4% vs. 42.3%, p = 0.04)[6]. Katzelnick demonstrated that use of these managers produced a greater proportion of patients in remission at 12 months (45.3% vs. 27.7%, p < 0.001) for a group of high utilizers [5]. Simon reported that specialist care management decreased the odds of persistent major depression at 6 months by more than half (OR = 0.45, 95%CI 0.24–0.86)[13]. Wells showed a greater proportion of responders at 6 months (64.4% vs. 54.4%, p = 0.005)[14]. In an older population using a depression specialist, Unutzer reported a greater proportion of responders at 12 months (45% vs. 19%, odds ratio 3.45, p < 0.001)[15]. In a rural population, Rost and colleagues demonstrated a greater reduction severity for depressed patients just beginning treatment (a decrease of 8.2 points more than usual care on a modified CES-D scale, 95% CI 0.2–16.1)[4]. Gensichen recently completed a meta-analysis of 13 studies and concluded that case management services improved the management of major depression in primary health care settings ; patients were more likely to achieve remission after 6–12 months (relative risk 1.39, 95% CI 1.30–1.48)[16]. The incremental cost-effectiveness ratios for specialist care managers ranged form $9,592 to $14,306 per quality-adjusted life-year [17].

Specialist care managers have varying responsibilities, which generally include many components–symptom assessment, medication support for the patient, monitoring to improve follow-up, communication with the primary care physician, patient education with self-management skills, decision support for the clinician, and the use of an information system. Despite the effectiveness of specialist care management, financial cost can be a major barrier to its implementation [18-22]. However, by managing multiple chronic conditions within primary care practices, a generalist care manager (GCM) may be both more accepted by the practices and more cost-effective than the specialist manager.

The North Carolina (NC) Division of Medical Assistance has been the leader among a handful of state Medicaid programs that have deployed expanded Primary Care Case Management (PCCM) models [23] for asthma and diabetes. These efforts have improved access to primary care services while reducing inpatient hospital services and emergency room costs [24]. Extension of these models to patients with depression could lead to improved care and lower costs for Medicaid programs, for two reasons. First, given the frequent comorbidity of diabetes and asthma with depression in primary care, GCMs who are able to manage all three conditions would be especially useful and efficient in a primary care setting. The prevalence of depressive illness appears to be at least twice as high among patients with diabetes [25] or severe asthma [26] as among control primary care groups, and diabetes complications [27] and worse asthma outcomes [28,29] are associated with depressive illness. Second, GCMs may be more readily incorporated into primary care practices than are specialist care managers, especially in small or rural practices, which might not have the critical mass of patients to support a specialist care manager.

In this context, GCMs can offer the same services to patients and physicians as their specialist care manager counterparts, but they can cover additional chronic conditions including asthma, diabetes, and/or depression. Such a role for a GCM for depressed patients has not been described previously. Because of the existence of care management for asthma and diabetes in NC and because of NC's leadership in PCCM, NC is an ideal setting in which to test the feasibility of applying GCM in this way. We designed a pilot program, PrimeCare, to demonstrate the feasibility of training GCMs and deploying them into primary care settings statewide.

## Methods

With a sample of Medicaid managed care patients, we piloted a randomized clinical trial comparing the benefits of a generalist care manager (GCM) for asthma, diabetes, and depression versus usual care (UC), in which care management was provided only for diabetes and asthma.

### Population and sample

Patients with depression were recruited from two primary care clinics in western North Carolina, the Mountain Area Health Education Center (MAHEC) in Buncombe County and the Hot Springs Health Program in adjacent Madison County. Buncombe County has an estimated population of 218,876, with 90% White and 7% Black. Four percent of the population is of Hispanic or Latino origin. Buncombe County is a mixed urban-rural area with 314.5 persons per square mile. The 1999 per capita income was $20,384, slightly higher than the state's per capita income of $20,307. Madison County has an estimated population of 20,256, with 98% White and 1% Black. Two percent of the population is of Hispanic or Latino origin. Madison County is a predominantly rural area with 43.7 persons per square mile. The 1999 per capita income was $16,076 [30]. MAHEC has 9 part-time attending physicians, 24 residents, and 1600 adult Medicaid patients; the Hot Springs program has 3 participating primary care physicians and 800 adult Medicaid patients.

We screened all adult Medicaid patients who visited the offices for medical services at the two primary care sites from July 2003 until February 2005 to identify those who were significantly depressed. We used the 9-item depression component of the Patient Health Questionnaire (PHQ-9) as the screening tool [31]. We provided screening feedback to physicians on all patients; for those who scored 10 or greater on this scale [32], the primary care physician verified the presence of major depression by clinical exam and determined previous histories of depression, experience with antidepressant medication and counseling services, and current wishes for treatment. Patients meeting selection criteria who were willing to begin or continue antidepressant medication were eligible for the pilot study.

We excluded from the study patients with bipolar disorder (as indicated by chart; patient self-report; positive response to either of the first two Structured Clinical Interview for DSM-IV (SCID) [33] questions for current or past manic episode; or history of Lithium use), psychotic symptoms (as indicated by chart; self-report; positive responses to the first SCID[33] question for persecutory delusions, auditory hallucinations, and visual hallucinations; or history of antipsychotic use), or active suicidal ideation requiring psychiatric admission (identified by PHQ-9 with further assessment as needed).

Consistent with other care management trials, [6,11-13] patients, rather than clinics, were randomized to the intervention, and randomization was stratified by clinic and by whether the patient was beginning or continuing antidepressant medication. Specifically, for each of the two sites, a list of 200 study ID numbers was generated, each with a random assignment to the treatment or control condition. Each random assignment was placed into a sealed envelope labeled with the corresponding ID number. The 120 lowest-numbered envelopes were reserved for patients with newly diagnosed depression, and the 80 highest-numbered envelopes were reserved in a separate box for patients already treated with antidepressant medication. Oncea patient was enrolled in the study, the care manager selected the appropriate box and opened the first envelope to determine the patient's treatment assignment. Randomization was done in blocks of four consecutive ID numbers so that allocation to the two conditions would be balanced over time.

All risks and benefits associated with PrimeCare participation were explained to participants, who provided written informed consent prior to study enrollment. The institutional review board at the University of North Carolina approved and monitored the protocol.

### Intervention

**Figure 1:**
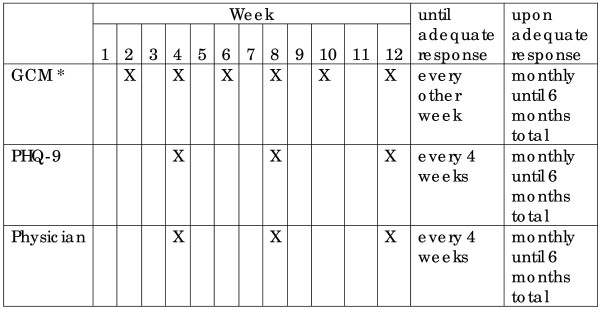
**Schedule for Follow-up Contacts with Patients**. *Discuss compliance with medication plan, barriers to adherence, depressive symptoms, level of functioning, side effects, questions/concerns, need for psychiatric referral or consultation with program psychiatrist. GCM is the Generalist Care Manager. PHQ-9 is the Patient Health Questionnaire-9 measure of depressive symptoms

The intervention consisted of GCM-provided care management for depression through: (1) monitoring of adherence to treatment guidelines and the presence of medication side effects, (2) routine follow-up by telephone or in-person visit every 2 weeks during the acute phase of treatment and every 4 weeks during the maintenance phase, (3) use of a case registry to monitor the course of illness and process of care, (4) patient education about depression and instruction in self-management skills and goals, (5) bi-weekly telephone supervision of the GCM (30 minutes) by the study psychiatrist and (6) coordination with the primary care physician and consultation with a psychiatrist as needed. Follow-up and PHQ-9 administration followed the schedule shown in Figure 1. This follow-up regimen was patterned after those utilized in published clinical trials and general recommendations [1,3,14].

GCMs who were already trained in care management skills for diabetes care and asthma care, received additional training in depression care management based on the training manual for depression specialists used in the IMPACT clinical trial of late-life depression in primary care [34]. The three GCMs were college graduates with specialization in mental health fields and had at least 5 years of care management experience before joining the Medicaid program.

Patients in the Usual Care (UC) group, whose physicians knew of their diagnosis and their placement in this UC group, received treatment and referral to mental health professionals as was consistent with the physicians' usual practices. These patients did not have access to the GCM for depression care, but patients already receiving diabetes and/or asthma care management continued to receive it. This design allowed us to assess whatever incremental benefit might be provided by the addition of depression care management.

A total of 45 patients were enrolled in the pilot program from July 2003 through February 2005, of whom 36 were followed up at 3 months and 34 followed up at 6 months.

### Measures

Standard demographic measures, including gender, ethnicity, marital status, education level, housing type, and age were collected from patients by the enrollment coordinator through self-report.

PHQ9: The Patient Health Questionnaire (PHQ-9) functioned as both a screen and a severity measure [35] and was administered at least 3 times (at baseline, 3 months, and 6 months) by physicians seeing the patients and/or by a research assistant blinded to the study arm of the patient. A cut point of 10 was used to detect moderate depression.

Hamilton Depression Index: The Ham-D is a well accepted research tool for measuring the severity of depression and response to treatment [36,37] and was collected by the enrollment coordinator at baseline and then by a research assistant blinded to the study arm of the patient at 3 and 6 months. A cut point of 14 was used to identify moderate depression.

Medical Outcomes Study Short Form-12 : The SF-12 is a commonly used measure of functional status and is divided into two scores, the mental Component Summary (MCS-12) and the Physical Component Summary (PCS-12). The MCS-12 and PCS-12 are scored based on 1998 US population norms, with a mean of 50 and a standard deviation of 10 [38]. The SF-12 was administered at baseline by the enrollment coordinator and at 6 months by the research assistant.

Physical comorbidity was measured by counting the number of 14 chronic physical conditions the subject reported at baseline, and was coded as two or more, one, or zero comorbidities [11].

Medication dosage was measured in three ways: chart abstraction was used to measure prescriptions documented by physicians, Medicaid claims data were used to measure prescriptions filled, and patient self-reports were used to measure medications taken. Medicaid data were available for all patients; chart and self-report data were 96% and 80% complete respectively.

Patients' perceptions of depression management were assessed by asking patients to rate on a 5-point scale from "strongly disagree" to "strongly agree" six items that were designed for this study: "(1) I understand that depression is a medical illness and treatment can make it better. (2) I can do things on my own that will help me better manage my depression. (3) I am comfortable with my doctor treating my depression with medications. (4) I understand the possible side effects that I might experience with the medicine for my depression. (5) The quality of care I have received for depression at this clinic has been good. (6)My care was explained to me in a way that I could understand." These data were collected by a research assistant blinded to the patient's treatment arm.

Components of GCM care: GCM case notes were abstracted by one of the authors (SL) to determine the nature and intensity of interactions with patients.

Value of GCMs for clinicians and staff: Four focus groups were held with 12 physicians and 9 clinical staff at the two sites (two groups of physicians and two groups of staff) to discuss integration of GCM into the primary care setting and perceived benefits and challenges

Time Study: We administered written questionnaires to personnel who participated in the study in order to measure average time spent interacting with the care manager before, during, and after the study period. The value of that time was calculated based on total compensation (wages+benefits).

Utilization data: Medicaid claims data were used to measure utilization of mental health and primary care services during the 12 weeks after study enrollment.

### Patient outcomes

Baseline measures included standard sociodemographic variables, the PHQ-9 score, the Ham-D score, the SF-12 score, and current use of antidepressant medication.

Patient outcomes at 3 and 6 months included % of patients with a ≥ 50% decrease in depressive severity (as measured by the PHQ-9 and Ham-D), % of patients with remission of depressive episode (as measured by PHQ-9 and Ham-D); adequacy of current dose and duration of treatment according to depression guidelines [39,40] ; changes in the SF-12 scores; and patients' perceptions of treatment. Utilization rates for medical and mental health services during the 12 weeks after enrollment were compared using t-tests.

### Data analysis

For the primary outcome variable, response status (PHQ-9 scores, Ham-D scores, SF-12 scores), we performed t-tests between GCM and UC in change from 0–3 months and change from 0–6 months. Chi-square tests were used to test the difference between GCM and UC in sociodemographic variables and proportion of patients using minimally adequate medication dose and proportion using moderately adequate medication dose and proportion using moderately adequate medication dose for at least 75% of each time period. For the evaluation of a pilot study, we set the alpha level at .15.

GCM notes and focus group comments were grouped and coded thematically. Predominant themes common across both clinic sites and types of interviewees (i.e., physicians and clinical support staff) and cited by all or most of the respondents are described as key, primary findings. Responses cited less often or responses that were not described by both types of informants or sites are listed as secondary findings. Responses offered by a single individual or small minority are not listed.

## Results

The study participants had a median age of 40.0 years and nearly all were female (95.6%); 31.8% were married or cohabiting. They were primarily White (62.2%), although 24.4% were Black and 13.3% had another race or ethnicity (mainly Hispanic or Russian/Ukrainian). Forty-five percent had less than a high school diploma and 54.6% lived in mobile homes. At baseline the median PHQ-9 score was 16.50 and median Ham-D score was 20.1(Table 1). In terms of clinical characteristics, nearly three fifths of the participants had previously been diagnosed with depression and three quarters had a serious pulmonary condition, diabetes, or hypertension. Other self-reported comorbid physical conditions were fairly common among this sample, and fully 84% of participants reported experiencing chronic pain. At baseline, the SF-12 means were about 1 SD below the population mean on mental health and 2 SD below the population mean on physical health. Compared to the general population of patients with depression, these statistics describe a relatively sick group with low socioeconomic status.

**Table 1:** Baseline sociodemographic and clinical characteristics (N = 45)

	n	Mean (SD) or n (%)		
		
		Usual care	GCM	Total
Age	45	41.2 (11.9)	38.1 (9.2)	39.7 (10.7)
Sex – female	45	22 (95.7%)	21 (95.5%)	43 (95.6%)
Race/ethnicity	45			
White only		14 (60.9%)	14 (63.6%)	28 (62.2%)
Black only		7 (30.4%)	4 (18.2%)	11 (24.4%)
Multiple races, other race, or Latino or Russian/Ukrainian ethnicity		2 (8.7%)	4 (18.2%)	6 (13.3%)
Marital status	44			
Never married		6 (26.1%)	6 (28.6%)	12 (27.3%)
Married or cohabiting		5 (21.7%)	9 (42.9%) *	14 (31.8%)
Separated, divorced, or widowed		12 (52.2%)	6 (28.6%) *	18 (40.9%)
Type of residence	44			
Mobile home		11 (47.8%)	13 (61.9%)	24 (54.6%)
Apartment or condominium		7 (30.4%)	4 (19.1%)	11 (25.0%)
House		5 (21.7%)	4 (19.1%)	9 (20.5%)
Education				
Years of schooling	44	11.8 (2.5)	11.5 (1.8)	11.7 (2.2)
Less than high school diploma	44	11 (50.0%)	8 (40.0%)	19 (45.2%)
Chronic medical conditions				
Number of conditions	44	3.2 (1.7)	4.2 (2.1) *	3.7 (1.9)
Major condition	44	17 (73.9%)	16 (76.2%)	33 (75.0%)
Asthma, emphysema, or chronic bronchitis	44	11 (47.8%)	11 (52.4%)	22 (50.0%)
Diabetes or hyperglycemia	44	4 (17.4%)	7 (33.3%)	11 (25.0%)
Hypertension	44	11 (47.8%)	9 (42.9%)	20 (45.5%)
Arthritis or rheumatism	44	9 (39.1%)	6 (28.6%)	15 (34.1%)
Cancer in last 3 years	43	2 (9.1%)	1 (4.8%)	3 (7.0%)
Gastrointestinal	44	5 (21.7%)	5 (23.8%)	10 (22.7%)
Hearing or vision loss	43	4 (18.2%)	8 (38.1%) *	12 (27.9%)
Heart disease	44	1 (4.4%)	2 (9.5%)	3 (6.8%)
Neurological condition	44	0 (0.0%)	5 (23.8%) **	5 (11.4%)
Pain	44	19 (82.6%)	18 (85.7%)	37 (84.1%)
Urinary tract	44	4 (17.4%)	11 (52.4%) **	15 (34.1%)
Clinical measures				
PHQ-9	43	15.9 (4.8)	17.3 (5.2)	16.5 (5.0)
HAM-D	43	19.7 (4.8)	20.6 (4.5)	20.1 (4.6)
SF-12 Mental Component Summary	39	42.8 (12.5)	40.7 (14.7)	41.8 (13.5)
SF-12 Physical Component Summary	39	30.3 (10.6)	28.4 (13.5)	29.4 (12.0)
Previously diagnosed depression	45	13 (56.5%)	13 (59.1%)	26 (57.8%)

* p < .15

** p < .05

PHQ-9 represents the Patient Health Questionnaire 9

HAM-D represents the Hamilton Depression Index score

SF-12 represents the Short Form-12 measure of functional status

SD represents standard deviation

The treatment and control groups were similar with respect to sociodemographic characteristics (Table 1). The GCM group as compared to UC group reported a slightly higher average number of self-reported comorbid chronic medical conditions and a higher proportion of neurological and urinary tract conditions.

### Clinical outcomes

Both study arms demonstrated progressive improvement in depression scores at 3 month and 6 month follow-up (Table 2). However, clinical outcomes of depression and functional status showed no significant differences between GCM and UC in change from 0–3 months or change from 0–6 months, except that UC improved more than GCM on the MCS-12 (Short Form 12 Mental Component Summary) from baseline to three months (p = .09; Table 2). Both arms improved substantially on physical health from baseline to 6 months (UC p = .003, GCM p = .04).

**Table 2:** Depression and functional status outcome score mean (SD) at baseline, three months, and six months

	Baseline		3 months		6 months	
	GCM	UC	GCM	UC	GCM	UC

PHQ-9	17.3 (5.2)	15.9 (4.8)	11.9 (7.1)	11.4 (5.7)	10.8 (5.9)	10.2 (5.9)
HAM-D	20.6 (4.5)	19.7 (4.8)	17.1 (6.7) *	13.5 (6.5)	14.4 (6.4)	12.4 (7.8)
MCS-12	40.7 (14.7)	42.8 (12.5)	38.8 (11.8) **	46.8 (10.6)	38.8 (12.2) *	45.3 (12.7)
PCS-12	28.4 (13.5)	30.3 (10.6)	33.6 (12.2)	35.7 (15.8)	37.4 (14.4)	39.3 (14.0)

SD represents standard deviation.

GCM represents the Generalist Care Manager arm of the study.

UC represents the Usual Care arm of the study.

PHQ-9 represents the Patient health questionnaire-9 measure for depression (range of 0–27).

HAM-D represents the Hamilton Depression Index score. For PHQ-9 (10 and above for moderate depression) and HAM-D (14 and above for moderate depression), higher scores indicate more depression.

MCS-12 represents the Mental component summary of the Short Form 12 functional status instrument.

PCS-12 represents the Physical component summary of the Short Form-12 functional status instrument. For MCS-12 and PCS-12 higher scores indicate better mental and physical health. All scores above or below 50 can be interpreted as above or below the general population norm. The standard deviations for each scale are equalized at 10.

* p < .15

** p < .05

The GCM proportion of patients using minimal/moderate adequate dose for at least 75% of each time period was greater for 10/12 individual variables (Table 3). Three of these tests (one for chart data and two for Medicaid data) had p < .15.

**Table 3:** Days of antidepressant medication "use" in past 30 days: proportion with > = 75% use

Source	Condition	3 months		6 months	
		
		minimal	moderate	minimal	moderate
Medicaid	GCM	50 *	32	36	18 *
	Usual Care	26	17	30	4
**Chart**	**GCM**	71 *	**48**	**52**	**33**
	**Usual Care**	**45**	**36**	**32**	**18**
Patient	GCM	72	44	44	28
	Usual Care	71	21	63	31

GCM represents the Generalist Care Manager arm of the study.

Medicaid represents claims data used to document prescriptions filled.

Chart represents chart abstraction to document medications written by physicians.

Patient represents patient self reported medication use.

Minimal represents the minimal dosing of antidepressants.

Moderate represents the moderate level of dosing of antidepressants.

* p < .15

### Patients' perceptions

Patients' perceptions of the care they received demonstrated slightly higher means (i.e., more positive responses) at 6 months than at 3 months, and were close to the scale point representing "Agree." For the items measuring patient perception of depression management, the lowest mean was for "there are things I can do myself" and the highest was for "care explained in a way I could understand." Patients agreed with most of the statements at both time points, and with respect to feeling that they had control in managing their depression, patients gave a neutral response at 3 months but moved closer to agreement by the 6 month point. At alpha = .15 there are no differences between patients with a history of depression and those with newly diagnosed depression. There are only two significant differences between GCM and UC: At 3 months the GCM patients had a higher mean on understanding side effects (3.5 > 3.1, p = .12) and at 6 months the GCM patients had a lower mean on quality of care (3.2 < 3.7, p = .10).

### Components of GCM Care

Record abstraction for the 19 GCM patients with 6 month follow up data documented that most of the interactions represented phone calls (40%), attempted phone calls (17%), or direct patient contact (15%). Aside from depression, comorbid conditions were the most common issues addressed (36%), then social issues (27%) and appointment reminders (14%). Only nine percent of GCM activities focused on adherence to depression meds and 10% on depression med side effects; 12% included a PHQ9 evaluation. A comparison of medians indicated that from baseline to the 6 month point, those with at least one chronic condition reported to the GCM other than depression (N = 11) had more contacts in person, by phone, and indirectly (i.e., with someone other than the patient). These increased numbers of contacts were focused more often on core issues of depression and chronic conditions than on social issues. For the 19 patients the GCMs recorded 873 service episodes, an average of 46 episodes per patient; and 59% involved one of the 11 patients with chronic conditions. Services for these patients were slightly more likely to be initiated by someone other than the care manager (5% vs. 1%), have side effects mentioned in the record (13% vs. 6%), or have PHQ9 mentioned (14% vs. 10%). Services for patients with these chronic conditions were less likely to have social issues mentioned in the record (21% vs. 34%). For patients with these chronic conditions, a slightly smaller proportion of services involved phone contact or attempted phone contact (53% vs. 61%) and more involved clinic or office visits (16% vs. 10%). Fewer of the contacts were with community agencies (0% vs. 8%) and more were with the patient or primary care provider (75% vs. 66%).

### Value of GCM for Clinicians

Focus group participants found the following GCM activities to be the most valuable: monitoring/tracking side effects of psychotropic medications, following up in initial weeks after diagnosis, scheduling/facilitating follow up appointments, making arrangements as needed to insure appointments were kept, and supporting clinical care teams as an extension of the staff. Items cited as having some value included being a supportive listener for patients, providing in-service training for nursing staff addressing social/human service issues, providing focused behavioral therapy, conducting home visits, and attending physician visits with the patients.

Focus group participants identified challenges to the utilization of GCMs. These included the need to have more information about the GCM's work with patients (preferably in the chart instead of through hallway conversations). They indicated that information should be succinct and easily accessible (e.g., in a standardized, checklist fashion). They requested that the GCM be on-site during all clinical hours and with sufficient capacity to respond during patient appointments. Physicians cited that separate reimbursement for follow up care from a payor such as Medicaid, and/or performance-based pay, may incentivize use of GCMs in a practice.

### Time Study

A total of 18 physicians and 9 staff completed the survey. Staff did not require more time to interact with GCMs, whereas physicians spent an average of 4 minutes more per week interacting with GCMs for depression care, as compared with the period after the study. The total impact of the time cost on physicians and other clinic staff is equal to 11% of the total GCM annual salary and benefits.

### Utilization Data

Based on Medicaid claims data, no difference existed between GCM and UC groups on visits to mental health specialists and primary care physicians for the 12 weeks after enrollment in the study. The means for mental health, primary care, and total visits respectively were 0.5, 3.1, and 3.7 for the GCM arm, and 0.7, 3.1, and 3.8 for the UC arm.

## Discussion

This pilot study was the first to start with a chronic disease care manager, already present in primary care and assisting in the management of asthma and diabetes, and provide further training and supervision in the management of depression. Our experience demonstrates that training and deployment of GCMs into primary care practices is feasible and provides some guidance for further development and testing of this management strategy

In this small pilot study, we did not see relative benefits for the GCM arm for the primary clinical outcomes. We found similar improvement in both treatment arms for depression and functional status outcomes, except for a greater improvement in general mental health status for the UC group. The clinical meaning of this one difference is unclear. Possible explanations for the general lack of differences include insufficient statistical power (due to the small sample size), patient non-adherence with the more aggressive treatment recommendations in the GCM arm, and a high quality of usual care making the benefit of the GCM arm harder to identify. Clinicians were aware of the study and had patients in both arms of the protocol; it is certainly possible that for patients enrolled in UC, the providers were more vigilant than usual in monitoring depression care themselves, thus diminishing the difference between the two study arms.

Another possibility is that GCM care for asthma and diabetes affected depressive symptoms. Seven (one third) of the patients in the UC group were receiving such services; the GCMs' presence in the lives of these patients may have been enough to improve their depressive symptoms or the GCMs may have encouraged these patients to follow the UC physicians' advice for depression, thus diluting the difference between the two study arms. Furthermore, poorly controlled asthma and diabetes can present with symptoms similar to depression such as fatigue, difficulty concentrating, and sleep disturbances. Care management that results in better asthma and diabetes control may improve these depressive symptoms. In addition, care managers working with asthmatic and diabetic patients focus on patients' knowledge of and correct use of glucometers and peak flow meters, and on the accurate recording of results. These patients may be more engaged in their medical and depression care than are patients without those medical illnesses. It seems possible that care management services for medical conditions could have improved depression outcomes in the UC group.

On the other hand, eleven (50%) of the GCM group were receiving care management services for asthma and/or diabetes, and the GCM group appeared to have more self-reported comorbid illnesses than the UC group; the care managers may have limited their time devoted to depression care management to focus on these patients' other illnesses. Data from the GCM case records support this assertion. In addition, the overall poor physical health of this Medicaid population (2 standard deviations below the norm for physical functioning-Table 2), the high rate of chronic pain (84%) reported, and the low self-efficacy (low score on "there are things I can do myself") present substantial impediments for GCMs and physicians to overcome.

Secondary outcomes, reflected by intermediate measures such as dosing of medications, were suggestive of benefit from use of GCMs. The results from a large number of tests, taken together, suggest that GCMs as compared to UC resulted in more written prescriptions of adequate medication dosages. Chi-square results were in this direction more often than would be expected by chance. This is more evident in the chart data than in the Medicaid and self-report data. This may indicate that GCMs influenced prescription writing by physicians more than they were able to influence prescription filling or medication use by patients.

Patient knowledge, attitudes, and satisfaction appear to have improved between the 3 month and 6 month follow-up points in both arms. It is not surprising that GCM patients with regular contact and education about these items had a better understanding of side effects than did UC patients at 3 months. However, the UC patients seem to have caught up by 6 months, perhaps as a result of education from their physicians and/or more time and experience with the medications. UC patients rated quality of care higher at 6 months than did GCM patients, a puzzling outcome that could represent some negative feelings in the GCM patients that physicians needed help from GCMs in order to provide care.

The GCM case notes illustrated the investment of time required, with an average of 46 interactions per patient. Although physicians valued the contributions of GCMs and spent a very small amount of extra time themselves to interact with GCMs, doctors were unwilling in their current fee for service environment to consider hiring and paying for GCMs themselves.

The patient population in this pilot study was weighted even more towards females (96%), minorities (40%), and patients with low educational status (45%) than other rural patient populations studied [4]. Along with the significant physical ailments, our population is quite disadvantaged and presents substantial challenges for physicians and health care systems in the design of intervention strategies to improve outcomes.

## Conclusion

A small pilot program designed and tested training materials, patient treatment algorithms and care management protocols for depression utilizing GCMs residing in two primary care practices. The NC Medicaid program adapted our antidepressant medication dosing algorithm and the GCM training and care protocol for implementation into selected primary care practices. From July 2005 through June 2007 the NC Medicaid program is reimbursing practices for providing GCM services for depressed patients and will be monitoring service utilization data and PHQ-9 patient outcomes. GCMs appear to be a feasible intervention for community medical practices; however, given the many questions raised by our data, we believe that a larger scale trial is warranted to test the appropriateness for Medicaid programs nationally.

## Competing interests

The author(s) declare that they have no competing interests.

## Authors' contributions

SL contributed to the conception and design, acquisition of data, and interpretation of data. BG contributed to the conception and design, acquisition of data, and interpretation of data. JM contributed to the conception and design, acquisition of data, and interpretation of data. NV contributed to the conception and design, acquisition of data, and interpretation of data. AE contributed to acquisition of data, analysis, and interpretation of data. MD contributed to the conception and design, acquisition of data, analysis, and interpretation of data. All authors read and approved the final manuscript.

## Pre-publication history

The pre-publication history for this paper can be accessed here:


